# Filtering high-throughput protein-protein interaction data using a combination of genomic features

**DOI:** 10.1186/1471-2105-6-100

**Published:** 2005-04-18

**Authors:** Ashwini Patil, Haruki Nakamura

**Affiliations:** 1 Institute for Protein Research, Osaka University, 3-2 Yamadaoka, Suita, Osaka 565-0871, Japan; 2 Department of Biology, Graduate School of Science, Osaka University, 1-1 Machikaneyama-cho, Toyonaka, Osaka 560-0043, Japan

## Abstract

**Background:**

Protein-protein interaction data used in the creation or prediction of molecular networks is usually obtained from large scale or high-throughput experiments. This experimental data is liable to contain a large number of spurious interactions. Hence, there is a need to validate the interactions and filter out the incorrect data before using them in prediction studies.

**Results:**

In this study, we use a combination of 3 genomic features – structurally known interacting Pfam domains, Gene Ontology annotations and sequence homology – as a means to assign reliability to the protein-protein interactions in *Saccharomyces cerevisiae *determined by high-throughput experiments. Using Bayesian network approaches, we show that protein-protein interactions from high-throughput data supported by one or more genomic features have a higher likelihood ratio and hence are more likely to be real interactions. Our method has a high sensitivity (90%) and good specificity (63%). We show that 56% of the interactions from high-throughput experiments in *Saccharomyces cerevisiae *have high reliability. We use the method to estimate the number of true interactions in the high-throughput protein-protein interaction data sets in *Caenorhabditis elegans*, *Drosophila melanogaster *and *Homo sapiens *to be 27%, 18% and 68% respectively. Our results are available for searching and downloading at .

**Conclusion:**

A combination of genomic features that include sequence, structure and annotation information is a good predictor of true interactions in large and noisy high-throughput data sets. The method has a very high sensitivity and good specificity and can be used to assign a likelihood ratio, corresponding to the reliability, to each interaction.

## Background

Protein-protein interactions in various organisms are increasingly becoming the focus of study in the identification of cellular functions of proteins. Though small scale experiments have contributed significantly to our knowledge of protein-protein interactions, the bulk of the data is available from high-throughput methods like yeast two hybrid (Y2H) and mass spectrometry of coimmunoprecipitated complexes (Co-IP) [1]. Such data is currently available for *H. pylori *[2], *S. cerevisiae *(baker's yeast) [3-6], *C. elegans *[7], *D. melanogaster *[8] and *H. sapiens *[9]. However, protein-protein interaction data obtained from high-throughput experiments is thought to have a large number of false positives i.e. interactions that are spurious or biologically irrelevant, and do not occur in the cell [10]. This fraction is estimated to be as high as 50% in yeast [1,11]. Since the false positives are unknown, there is no consensus on which interactions from these data sets should be used in prediction studies. Studies that use all the interactions run the risk of predicting spurious ones [12], while those that completely ignore the high-throughput data are limited by the amount of data from small scale experiments[13]. For some high-throughput studies, the authors specify the reliable interactions as 'high confidence' or 'core' interactions [4,7,8] which have fewer false positives but which do not take into account those low confidence interactions which are known to be true. Hence it is important to quantify the reliability of these interactions and identify the true positives i.e. interactions that actually occur in the cell.

Several methods have been previously used to identify true interactions from high-throughput experimental data in yeast. Sequence homology was used by Deane *et al*. [14] in the form of a paralogous verification method (PVM) whereby an interaction in yeast is judged to be true if the concerned proteins have paralogs that interact as well. But these results are limited by the number of proteins that have known paralogs. They also used similarity in gene expression profiles to identify true positives [14]. Structurally known interactions were used by Edwards *et al*., who compared experimental interactions found in RNA polymerase II, Arp2/3 complex and the proteasome with those observed in the 3D structures [15]. Though this method has a high reliability, it is limited by the number of structures available in Protein Data Bank (PDB) [16]. von Mering *et al*. found the interactions in yeast that are observed in more than one high-throughput experiment to estimate the fraction of true positives [1]. The results obtained were surprisingly small in this case due to inherent biases in different experimental methods. Database annotations have been used by Sprinzak *et al*. in the form of co-localization data of the interacting proteins and their cellular role to estimate the number of true positive interactions in yeast [11]. However, not all model organisms have well annotated genomes. Interaction network topology is another means of identifying true interactions. Saito *et al*. used an interaction generality measure (IG2), based on the topological properties of the interaction network, to assess the reliability of an interaction [17]. Bader *et al*. used screening statistics and network topology to quantify the confidence of each interaction [18]. Though these methods have a high specificity (low false positive rate), they have low sensitivity (low true positive rate), since the number of proteins with more than one interaction partners is relatively few.

Since none of the methods give a good performance (high sensitivity and specificity) by themselves, it follows that a combination of methods would perform better. Jansen *et al*. have shown that a combination of genomic features results in a more accurate prediction of the yeast protein interaction network [19]. In this study, we use a similar approach of combining various genomic features using naïve Bayesian networks to predict the true interactions in high-throughput data sets.

In selecting the genomic features to be used in our model, we decided to combine sequence, structure and database annotation information about the interaction. Sequence information was incorporated through homologous interactions. We used our Homologous Interactions (HINT) database [20] to obtain homologs for all high-throughput interactions [21]. Structure information was incorporated in the form of interacting Pfam domains [22] found in the PDB. We used the 3did database to obtain a list of such Pfam domains [23]. Database annotation information was used in the form of Gene Ontology (GO) terms used to describe the interacting proteins [24].

We computed the reliability of each feature using likelihood ratios and combined their evidence using naïve Bayesian networks in order to predict the true interactions from high-throughput data sets. Bayes' rule provides a good method to estimate posterior odds of an event in the presence of prior evidence [25]. Bayesian approaches have also been used frequently in the past to calculate the reliability or to assign probabilities to protein-protein interactions [15,19,26].

In this study, we show that an interaction can be judged to be true if either or all of the following are true:

1. the interacting proteins have homologs that interact,

2. the interacting proteins each have a Pfam domain found to interact with the other in PDB and,

3. the interacting proteins have at least one identical GO annotation.

We used protein-protein interaction data from the Database of Interacting Proteins (DIP) [27] (July 2004 release) and IntAct [28] (September 2004 release). We prove our hypothesis first in yeast by estimating likelihood ratios for high-throughput interactions based on the number of known true positives and false positives using Bayesian network approaches. Based on these results, we estimate the number of true positives in the high-throughput data sets of *S. cerevisiae*, *C. elegans*, *D. melanogaster *and *H. sapiens*. The results can be searched at and downloaded from our website [29].

## Results

### Calculating the reliability of each genomic feature

We used protein-protein interactions from high-throughput data sets for yeast as our test set to calculate the reliability of each genomic feature (see Methods). Of these 12,674 interactions, we chose a set of 3464 interactions as our gold standard – 1479 as the positive gold standard and 1985 interactions as the negative gold standard (see Methods). Our goal was to maximize the interactions identified in the gold positive set (high sensitivity) and at the same time minimize the number of interactions identified in the gold negative set (high specificity).

In these interactions, we identified all those that had homologous interactions. The true positives (TP) were those interactions with homologs that were in the gold positive set, and the false positives (FP) were those that were in the gold negative set. Using these values, we calculated the likelihood ratio (L) for the genomic feature of 'homologous interactions' (see Methods). Similarly, we calculated the likelihood ratios for the other two genomic features – interacting proteins with at least one identical GO annotation and interacting proteins having one of 2 Pfam domains known to interact in PDB. We also calculated the likelihood ratio for the absence of genomic features.

**Figure 1:**
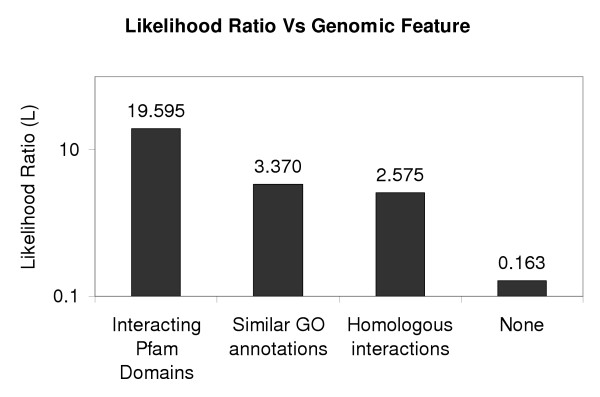
Likelihood ratios for genomic features.

Figure 1 shows the likelihood ratios calculated. Likelihood ratio (L) expresses the reliability of each genomic feature. An L > 1 indicates the ability of the genomic feature to identify more true positives than false positives. As seen in Figure 1, all the genomic features have L values greater than 1. The absence of any genomic feature to support the interaction results in L < 1. This indicates that in the absence of any support from the selected genomic features, the interaction is more likely to be a false one. Interacting Pfam domains in the interacting proteins gives the highest L showing that interactions with evidence from structural data have the highest reliability. This is followed by the L values of similar GO annotations for interacting proteins and the presence of homologous interactions respectively.

### Using naïve Bayesian Networks to combine the evidence of genomic features

We used naïve Bayesian networks to combine the evidence of each genomic feature for a particular interaction. Since naïve Bayesian networks require that the genomic features be conditionally independent of each other, we calculated the Pearson's correlation coefficient for a pair of genomic features to ascertain their independence (see Methods). We then combined the evidence of each interaction by simply multiplying the L values of each genomic feature found for the interaction. Thus, to each interaction in the gold set of 3464 interactions, we assigned an L value based on the genomic features it had. An L value greater than 1 represents higher posterior odds of an interaction being true than prior odds (see Methods). Hence all interactions with an L value greater than 1 were predicted as true. Table 4 shows the L values obtained for each possible combination of genomic features supporting an interaction. For instance, an interaction that is supported by the presence of all 3 genomic features has the highest L value, thus having the highest probability of being true.

### Assessing the accuracy of the predictions in yeast

**Figure 2:**
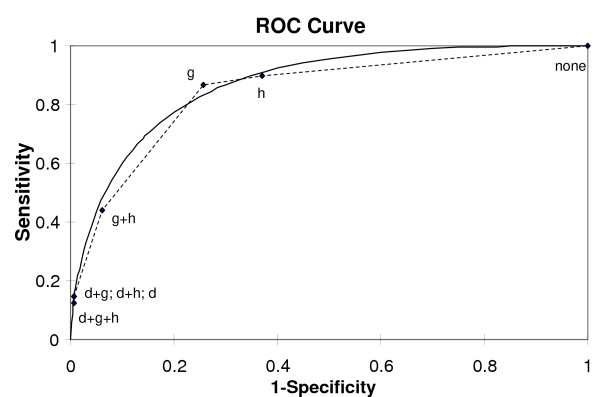
**ROC curve for the combination of genomic features using 10-fold cross validations. **The dotted line shows the empirical ROC curve, while the solid line shows the fitted ROC curve (obtained using JROCFIT). Each point on the ROC curve corresponds to sensitivity and specificity for one or a combination of more than one genomic features. d: interacting Pfam domains; g: similar GO annotations; h: homologous interactions; none: no genomic features. More than one genomic features are indicated by listing the features separated by a '+' sign.

To assess the accuracy of our method, we identified the number of predicted true interactions in the gold positive set and those in the gold negative set respectively. We conducted 10-fold cross-validation on the limited set of yeast high-throughput interactions to calculate the sensitivity and specificity of the method. Figure 2 shows the receiver operating characteristic (ROC) curve for our method. Each point on the ROC curve denotes the sensitivity and specificity obtained on the inclusion of interactions with a lower L value. A particular L value is associated with a specific combination of the 3 genomic features (Table 4). Thus, including the interactions supported by the presence of all 3 genomic features (d+g+h) in the results gives a sensitivity of 12.3% and a specificity of 99.4%. On further including interactions supported by interacting Pfam domains and similar GO annotations (d+g), the sensitivity rises to 14.5% and the specificity marginally decreases to 99.3%. As interactions supported by each individual feature or other combinations of features that have an L > 1, are included in the results, the sensitivity increases at the cost of specificity. Thus our method predicts interactions, which are supported by at least one of the 3 genomic features, to be true with a sensitivity of 89.7% and a specificity of 62.8%.

### Predicting true interactions in all high-throughput data sets

**Figure 3:**
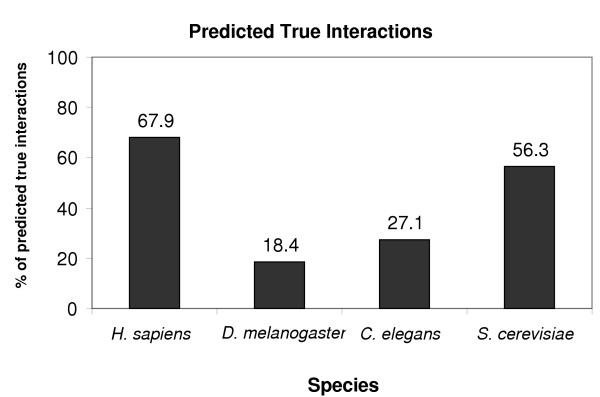
Percentage of interactions predicted true across different high-throughput data sets.

We used our method to assign L values to all interactions in three other high-throughput data sets for *C. elegans*, *D. melanogaster *and *H. sapiens *[7-9]. We also assigned L values to the interactions in yeast high-throughput data sets [3-6] that were not part of the gold standard. We predicted all interactions with L > 1 as true interactions. Table 5 shows the distribution of the predicted true interactions across different L values for each species. Figure 3 shows the percentage of interactions predicted as true in the high-throughput data sets of each species.

**Figure 4:**
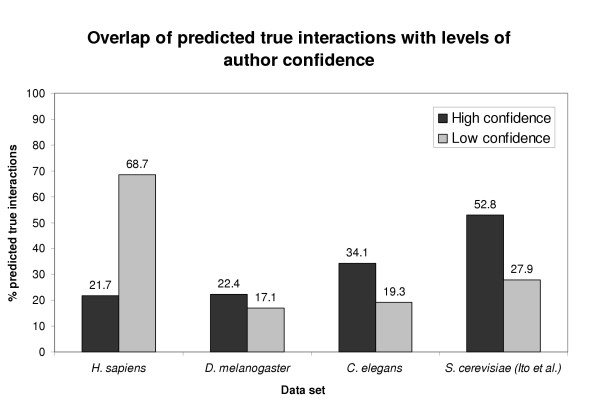
Percentage of interactions predicted true in high and low confidence interactions across different high-throughput data sets.

Authors of the high-throughput data sets usually assign a confidence level to interactions. Those interactions that are either reconfirmed experimentally or have a high probability of being true based on some statistical method are deemed as high confidence with the rest being low confidence interactions. We tested the overlap between our predicted true interactions and the high and low confidence data sets given by the authors. As seen in Figure 4, more high-confidence interactions are predicted as true in all data sets, except in *H. sapiens *[9]. For instance, 52.8% of the high confidence interactions in yeast are predicted to be true by our method, as opposed to 27.9% of the low confidence interactions.

### Some validated predictions

**Figure 5:**
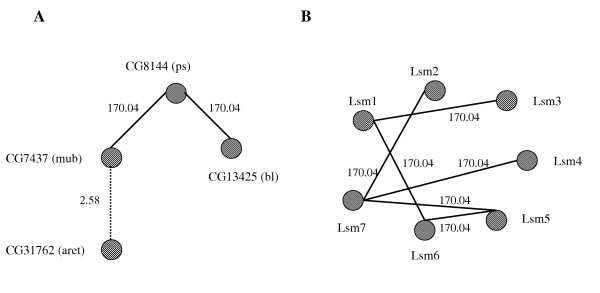
**Some low confidence interactions predicted to be true by our method and confirmed by other publications. **The Likelihood ratio for each interaction is indicated. Interactions with a Likelihood ratio greater than 100 are shown with a solid line, while those with a Likelihood ratio less than 10 are shown with a dashed line. (A) Interactions between proteins co-regulating the alternative splicing of *Dscam *exon 4 in *D. menalogaster*. (B) Interactions between proteins in the Lsm1-7 complex in *S. cerevisiae *confirmed by similar interactions found in *H. sapiens*.

Figure 5 shows two instances where our method predicts low confidence interactions to be true. Figure 5A gives the interactions between the proteins ps, mub, bl and aret. These proteins have all been recently shown to co-regulate the alternative splicing of *Dscam *exon 4 in *D. melanogaster *[30]. Figure 5B shows the interactions between the Lsm proteins in the mRNA degradation process in yeast that were predicted to be true by our method. These interactions were later confirmed by similar ones in the human mRNA degradation process [31].

## Discussion

We present here a method to identify the true interactions in high-throughput protein-protein interaction data sets using a combination of three genomic features. We used the likelihood ratio (L) to evaluate the accuracy and reliability of each genomic feature. We combined the evidence from each genomic feature using naïve Bayesian networks. Our method gives a sensitivity of 89.7% which is higher than any of the other methods used so far. Our method also has a good specificity at 62.9%. We chose the three genomic features to maximize the inclusion of all aspects of information about the interactions.

Structure information was incorporated through Pfam domains found to interact in PDB structures in the 3did database. As would be expected, this feature has the highest accuracy and reliability as shown by its high L value (Figure 1). As seen in the ROC curve (Figure 2), this genomic feature gives the lowest number of false positives (high specificity). However, the number of true positives (sensitivity) is limited by the small number of complex structures in PDB that can be used to identify interacting Pfam domains. The sensitivity will significantly improve as the number of structures in PDB increases.

Database annotations were included through the use of GO annotations of the interacting proteins. This feature shows the second highest reliability (Figure 1). It is also able to identify the maximum number of true positives. Indeed, Lin *et al*. have recently shown that GO annotations are the dominant contributors in predicting protein-protein interactions [32]. As the number of annotated proteins increases, this method promises to be useful in filtering interaction data.

Sequence information was included in the form of homologous interactions found using the HINT database. Homologous interactions do not give the reliability expected (Figure 1), perhaps because they are not limited to orthologous or paralogous interactions. However, it is the only feature that does not require any protein annotations and is useful in identifying true interactions of un-annotated or hypothetical proteins. Methods based on network topology [17,18] are also independent of protein annotations and would be a useful addition to the genomic features. However, we have not considered it in the current study.

Though evidence from each feature can independently predict an interaction to be true, a combination of 2 or more features performs better (Table 4). For instance, the combination of interacting Pfam domains and similar GO annotations (d+g) or interacting Pfam domains and homologous interactions (d+h), has a higher L value than either of the features independently. Both combinations increase the sensitivity without much compromise in the specificity. Similarly, a combination of similar GO annotations and homologous interactions (g+h), predicts an interaction to be true with a higher probability than each feature independently. This combination too adds to the sensitivity with only a slight decrease in the specificity. Surprisingly, evidence from interacting Pfam domains (d) performs better than that of the combination of the other two features (g+h), highlighting the importance of the incorporation of structural evidence.

Due to the absence of information about non-interacting proteins, we prepared our gold negative set from proteins that have different subcellular localizations. However, some interactions are transient with interacting proteins residing in the same sub-cellular compartment for only a small fraction of their life time. As a result, some of the interactions in the gold negative set are actually true. Thus, the specificity of our method is probably higher than 62.9%.

Using the evidence of the three genomic features, we predicted the number of true interactions in various high-throughput data sets. Our prediction of 56.3% true interactions in yeast high-throughput data sets is in conformance with the previous estimates of the number of false positives in these data sets [1,11]. However, yeast Y2H [3,4] and Co-IP data sets [5,6] show very different numbers of true positives independently – 37% and 73% respectively (data not shown). The *D. melanogaster *data set [8] shows a very low rate of true positives. One reason could be that this experiment was performed using most of the predicted transcripts in the *D. melanogaster *genome, including biologically irrelevant ones. The *C. elegans *data set [7] includes a higher percentage of true positives than *D. melanogaster*, perhaps because the experiment was performed only on a restricted set of predicted proteins related to multi-cellular functions. The *H. sapiens *data set [9] shows the highest number of true positives at 67.9%. This data set was obtained from a study that focused on the identification of putative protein complexes in the TNF-α/NF-κB signal transduction pathway using Co-IP [9]. There are two possible reasons for the high number of true positives. Firstly, the choice of proteins from a specific signal transduction pathway precludes many random interactions between proteins of unrelated functionality. Secondly, the Co-IP approach to the identification of protein complexes, and thus interactions, is known to have a low false positive rate of around 20% [1,9], in comparison to Y2H approaches. This is also reflected in the prediction of a larger number (73%) of true positive interactions in the Co-IP data sets of yeast[5,6] by our method.

We also studied the overlap of the predicted true interactions with the high-confidence and low-confidence interactions as given by the respective authors. Though the number of interactions predicted in high-confidence data sets is higher, 17–28% of the interactions in low-confidence data sets are also predicted to be true, except in the *H. sapiens *data set. This shows that some low-confidence interactions can be biologically relevant. When compared to other data sets, the predicted true interactions in *H. sapiens *data set show a much higher overlap (68.7%) with the low confidence interactions. This is because the high confidence data set given by Bouwmeester *et al*. primarily focuses on interactions, novel or otherwise, that are most likely to be a part of the signalling cascade triggered by TNF-α [9]. Hence, interactions of proteins that are also part of other systems, like the cell cycle, are not included in this high confidence data set. These include interactions of nucleasome assembly proteins and MCM proteins, among others. Other interactions which have been filtered out include those of frequently copurified proteins like the Heat Shock Proteins. In order to limit their interaction map to the TNF-α/NF-κB signal transduction pathway, the authors have chosen a very stringent statistical criterion to identify the interactions of proteins that are expressed well above their normal levels on being triggered by TNF-α [9]. As a result, the high confidence data set as given by Bouwmeester *et al*. forms only 10% of the total interactions identified in their study, while our method predicts a large number of low confidence interactions to be true.

We were also able to confirm several of the low confidence interactions, that were predicted as true, in literature using iHOP [33]. In fact, most of the interactions of the Lsm proteins, shown in Figure 5B, are found in iHOP. Several interactions from the human dataset are also found in iHOP and the Human Protein Reference Database [34]. Among others, these include the low confidence interactions of the C-Rel proto-oncogene with itself, NK-κB p105 subunit, NF-κB p100/p49 subunits, Heat shock cognate 71 kDa protein and NF-κB beta inhibitor. This further reiterates the biological relevance of a large number of low confidence interactions.

## Conclusion

In this study, we show that a combination of genomic features that includes sequence, structure and annotation information, can be used to identify true interactions from high-throughput protein-protein interaction data sets. We use likelihood ratios to assess the reliability of each genomic feature and combine their evidence using naïve Bayesian networks. We provide a likelihood ratio for each predicted true interaction based on the evidence that supports it. Our method has a high sensitivity and a good specificity. The results of our study are available on our website [29]for search and download.

## Methods

### Yeast high-throughput data sets

Table 1 shows the number and type of interactions from the 4 yeast high-throughput data sets used. Data inferred from mass spectrometry of coimmunoprecipitated complexes (Co-IP) is converted to binary interactions using the spoke model (the spoke model has been previously shown to be more reliable than the matrix model [18,35]).

### Gold standard data sets

The gold standard positive data set consisted of:

1. all physical interactions from MIPS [36] yeast two-hybrid data (excluding interactions from Uetz *et al*. [3] and Ito *et al*.[4]),

2. MIPS complexes data (excluding complexes from Gavin *et al*. [5] and Ho *et al*.[6]),

3. small-scale yeast-two hybrid experimental data from DIP and IntAct and,

4. interactions found in more than one high-throughput data sets.

Table 2 shows the number and type of interactions from each data set. For this study, the gold standard positives are limited to those found in the yeast high-throughput data sets i.e. 1479 interactions, instead of all possible gold standard positives. This is because the aim is to identify the true protein-protein interactions in high-throughput data setsin yeast, as opposed to predicting *all *true protein-protein interactions in yeast.

The gold standard negative data set is derived from protein localization data in yeast cells [37]. Proteins that do not exist in the same sub-cellular compartment are assumed to be non-interacting since the majority of the interactions occur between proteins in the same sub-cellular compartment [19,38,39]. As with the gold standard positive, the gold standard negative data set is also limited to those interactions found in the yeast high-throughput data sets i.e. 1985.

### Genomic features

In order to predict true interactions, we identified those that have at least one of the following genomic features based on:

1. Homologous interactions – Using our HINT database [21], we identified all interactions from high-throughput data sets that had homologous interactions, including orthologous or paralogous interactions. An interaction is deemed as homologous to a given interaction when each of its interacting proteins has homologs that are found to interact in DIP or IntAct. Homologs of interacting proteins are identified by HINT using PSIBlast with 5 iterations and an E-value cut-off of 10^-8^.

2. GO annotations – Using data from the GO database [24], we identified all interactions from high-throughput data sets where the interacting proteins shared at least one GO term, since interacting proteins generally share a common function [39].

3. Interacting Pfam domains – We identified interactions in high-throughput data sets, where each of the interacting proteins had one of the two Pfam domains that were found to interact in PDB structures by the 3did database [23].

### Correlation between genomic features

The correlation between each genomic feature was calculated using Pearson's correlation coefficient for 100 random interactions from the high-throughput data sets. The significance of each correlation coefficient was tested using a t-test with 98 degrees of freedom. Table 3 shows the correlation coefficients, t values and the probability. All the genomic features were found to be independent of each other.

### Bayesian networks

Bayesian networks can be used to combine evidence from different sources and calculate the posterior odds of an event based on prior evidence [25]. The relation between the posterior odds and prior odds of finding a true interaction is given by Bayes' rule as follows:

O_posterior _= L(g_1_, g_2_, g_3_,..., g_N_) O_prior_,    (1)

where g_1_, g_2_, g_3_,....., g_N _are genomic features of an interaction,

O_prior _= prior odds of an interactions being true,

O_posterior _= posterior odds of an interaction with N genomic features being true,

L(g_1_, g_2_, g_3_,..., g_N_) = likelihood ratio of an interaction with genomic features.



where P (true) = probability of an interaction being true.



where P(true|g_1_, g_2_, g_3_,..., g_N_) = probability of an interaction with N genomic features being true.

From equation (1), the likelihood ratio is,

,

where P (g_1_, g_2_, g_3_,..., g_N _|true) = probability of a true interaction having N genomic features.

If the N genomic features, g_1_, g_2_, g_3_,......, g_N_, are conditionally independent, then the resulting Bayesian network is called a naïve Bayesian network and its likelihood ratio can be given as the product of the likelihood ratios for each feature:







where T = all true interactions (gold standard positives),

F = all false interactions (gold standard negatives),

TP_i _= number of true interactions in the high-throughput data set with the i^th ^feature

FP_i _= number of false interactions in the high-throughput data set with the i^th ^feature

For any organism, L(g_1_, g_2_, g_3_,..., g_N_) > 1, results in O_posterior _> O_prior_. This is because, in equation (1), O_prior _is a constant and depends on the number of interactions in any organism. Hence, O_posterior _is directly proportional to L(g_1_, g_2_, g_3_,..., g_N_). Thus, the posterior odds of an interaction being true, if it has one or more genomic features, increases as L(g_1_, g_2_, g_3_,..., g_N_) increases i.e. larger the L(g_1_, g_2_, g_3_,..., g_N_), the higher are the odds of an interaction being true.

### ROC curve analysis

A Receiver Operating Characteristic (ROC) curve is a graphical representation of the accuracy of a test and expresses the trade-off between the sensitivity and the specificity of the test [40]. Sensitivity of a test is defined as the ability to identify a true positive in a data set. Specificity is defined as the ability to identify a true negative in a data set.







where TP = number of true positives,

TN = number of true negatives,

FP = number of false positives,

T = total number of positives,

F = total number of negatives.

The ROC curve is plotted with the Sensitivity on the Y-axis and (1-Specificity) on the X-axis. The smooth ROC curve is plotted using JROCFIT [41].

### Cross-validation

Since the training set (data set used to calculate the likelihood ratios) and the test set (data set used to calculate the sensitivity and specificity) are the same yeast high-throughput data set, we used 10-fold cross-validation to assess our predictions. We divided the positive and negative gold standards into 10 approximately equal sets. We used 9 of these to calculate likelihood ratios for each genomic feature. Then we identified the true positives and false positives in the remaining set using these likelihood ratios. We did this in turn, so that each of the 10 sets was a test set and the remaining 9 sets were training sets. We then summed the number of true positives and false positives across all the 10 test sets to obtain the Sensitivity and Specificity and plotted the ROC curve.

## Authors' contributions

HN and AP conceived of the study. AP performed the data collection, data analysis, web site preparation and drafted the manuscript under the guidance and supervision of HN. All authors read and approved the final manuscript.

## Figures and Tables

**Table 1:** Yeast high-throughput data sets

Data set	Interactions	Type
Uetz *et al*. [3]	1438	Y2H
Ito *et al*. [4]	4449	Y2H
Gavin *et al*. [5]	3757	Co-IP (spoke model)
Ho *et al*. [6]	3618	Co-IP (spoke model)
Total unique interactions	12674	Binary

Y2H: Yeast two-hybrid; Co-IP: Mass Spectrometry of coimmunoprecipitated complexes, converted to binary interactions using the spoke model.

**Table 2:** Sources of Gold Standard Positive yeast protein interaction data

Data set	Interactions	Type
MIPS interactions	574	Y2H
MIPS complexes	490	Co-IP (matrix model)
Small scale interactions from DIP and IntAct	110	Y2H
More than one high-throughput data sets	305	Y2H ([3, 4]) Co-IP (spoke model) [5, 6]
Total	1479	Binary

Y2H: Yeast two-hybrid; Co-IP: Mass Spectrometry of coimmunoprecipitated complexes, expanded by spoke or matrix model as indicated.

**Table 3:** Correlation coefficients of the genomic features for 100 random interactions

Genomic Features	r	t(98)	p-value
Homologous Interactions – Similar GO annotations	-0.12605	-1.2579	0.2401
Homologous Interactions – Interacting Pfam Domains	0.022501	0.222802	0.8826
Similar GO annotations – Interacting Pfam Domains	-0.01817	-0.17988	0.2868

r: Pearson's correlation coefficient; t(98): t-test with 98 degrees of freedom; p-value: probability. Since the p-value for all t-tests is greater than the significance level of 0.05, the null hypothesis, that the genomic features are not correlated, is accepted.

**Table 4:** Likelihood ratio, sensitivity and specificity for the combination of different genomic features

Genomic Feature(s)	Likelihood ratio (L)	Sensitivity (%)	Specificity (%)
d + g + h	170.052	12.3	99.4
d + g	66.031	14.5	99.3
d + h	50.463	14.7	99.2
d	19.595	14.8	99.2
g + h	8.678	44.1	94.0
g	3.370	86.7	74.3
h	2.575	89.7	62.9
none	0.163	100	0

d: interacting Pfam domains; g: similar GO annotations; h: homologous interactions. More than one genomic features are indicated by listing the features separated by a '+' sign.

**Table 5:** Number of interactions in different ranges of likelihood ratios for high-throughput data sets of various species

Likelihood ratio (L)	*H. sapiens*	*D. melanogaster*	*C. elegans*	*S. cerevisiae*
0 – 1	541	16655	2925	5534
1 – 10	733	3119	852	5810
10 – 100	362	367	139	824
100 – 1000	50	260	99	506
Total	1686	20401	4015	12674

All interactions with a Likelihood ratio > 1 are predicted as true.
